# Sphingolipids and Diagnosis, Prognosis, and Organ Damage in Systemic Lupus Erythematosus

**DOI:** 10.3389/fimmu.2020.586737

**Published:** 2020-09-25

**Authors:** Olivia C. Harden, Samar M. Hammad

**Affiliations:** ^1^College of Medicine, Medical University of South Carolina, Charleston, SC, United States; ^2^Department of Regenerative Medicine and Cell Biology, Medical University of South Carolina, Charleston, SC, United States

**Keywords:** sphingolipid, sphingomyelin, ceramide, sphingosine, sphingosine 1-phosphate, lipidomics, sphingolipidomics, lupus

## Abstract

Systemic lupus erythematosus (SLE) is a chronic autoimmune disease that involves multiple organs and disproportionality affects females, especially African Americans from 15 to 44 years of age. SLE can lead to end organ damage including kidneys, lungs, cardiovascular and neuropsychiatric systems, with cardiovascular complications being the primary cause of death. Usually, SLE is diagnosed and its activity is assessed using the Systemic Lupus Erythematosus Disease Activity Index (SLEDAI), Systemic Lupus International Collaborating Clinics Damage Index (SLICC/ACR), and British Isles Lupus Assessment Group (BILAG) Scales, which unfortunately often occurs after a certain degree of systemic involvements, disease activity or organ damage already exists. There is certainly a need for the identification of early biomarkers to diagnose and assess disease activity as well as to evaluate disease prognosis and response to treatment earlier in the course of the disease. Here we review advancements made in the area of sphingolipidomics as a diagnostic/prognostic tool for SLE and its co-morbidities. We also discuss recent reports on differential sphingolipid metabolism and blood sphingolipid profiles in SLE-prone animal models as well as in diverse cohorts of SLE patients. In addition, we address targeting sphingolipids and their metabolism as a method of treating SLE and some of its complications. Although such treatments have already shown promise in preventing organ-specific pathology caused by SLE, further investigational studies and clinical trials are warranted.

## Introduction

Systemic lupus erythematosus (SLE) is a systemic, chronic autoimmune disease that could manifest in any organ system. The cause of SLE is unknown; however, a combination of genetic, environmental and hormonal factors seem to play a role in its evolution. SLE most commonly (65%) presents in minority women of childbearing age (15–44) (1); 20% of individuals are diagnosed with SLE before age 15 and 15% after 55 years of age (2, 3). According to the CDC, Hispanic, Asian, American Indians/Alaska Natives, and African American women are disproportionality impacted by SLE in comparison to White women (4). The prevalence of SLE in the United States is 20–150 per 100,000 with the prevalence varying by race (5, 6). Prevalence rates vary greatly with African American women at 406/100,000 and White women at 164/100,000 (5, 6).

Lupus is associated with classic symptoms of fatigue, fever, myalgia, and weight change that are collectively known as constitutional symptoms and affect 50% or greater of SLE patients (7). In addition, arthritis and arthralgia affect over 90% of SLE patients. Importantly, SLE can lead to end organ damage including kidneys, lungs, cardiovascular, and neuropsychiatric systems, with cardiovascular complications being the primary cause of death (7, 8).

The cost of treatment for SLE vary significantly based upon the severity of disease. In a study conducted by Clarke et al. (9), the mean 12 month-adjusted cost of treatment for mild SLE and moderate/severe SLE was determined as $28,298 and $47,542, respectively. Chronic diseases are expensive to treat and severely lower the quality of life of the patients as well as their life span; therefore, diagnosis and early treatment of SLE would ensure healthy life years and save money. There is certainly a pressing need for diagnostic tests and criteria that can be used prior to the development of any life-altering SLE symptoms. In this review, the role of lipidomics, more specifically sphingolipidomics, is discussed as an added potential tool to fill this void. There is emerging evidence that imbalances in sphingolipid metabolism and alterations in sphingolipid levels in the circulation may be present prior to the onset of classic SLE symptoms. Thus, sphingolipid measurements could have the potential to be used as an early predictor of SLE and its comorbidities, and may lead to improved diagnosis, prognosis, and treatment of the disease.

### Sphingolipids: Structure and Function

Sphingolipids are a key component of cells and have traditionally been considered structural in function due to their presence in cellular membranes. However, we now know that they are bioactive molecules that function as signaling molecules regulating cellular processes including apoptosis, proliferation, growth, and other vital cellular processes (10, 11).

**FIGURE 1:**
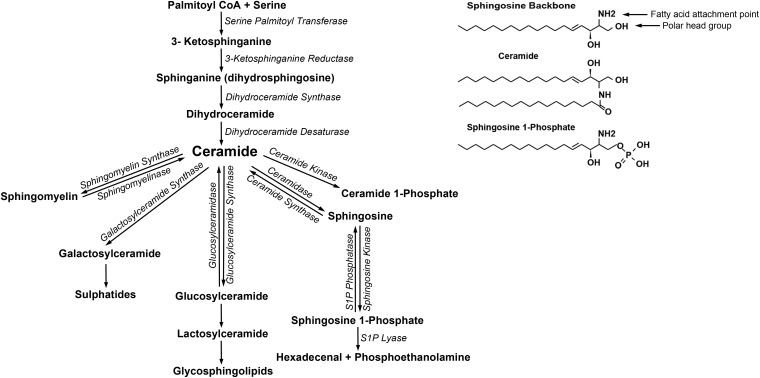
Sphingolipid structure and metabolic pathway.

Sphingolipids are a class of lipids composed of a sphingosine backbone, which have attachment points at the alcohol group at carbon 1 and a fatty acid attachment point on the carbon 2 (Figure 1). When a long-chain amino alcohol is attached to the polar alcohol group, a sphingolipid is formed (12, 13). Ceramides are formed when an N-acetyl fatty acid is attached to sphingosine. Sphingomyelin (SM), one of the most abundant sphingolipids, is composed of the sphingosine base, a fatty acid chain and phosphocholine attached forming the polar head group. When a sugar is attached to the polar head group, the molecule becomes a glycosphingolipid (12, 13).

Sphingolipids can be generated *de novo* starting with the condensation of the amino acid serine and palmitoyl CoA via the enzyme serine palmitoyltransferase to form 3-ketosphinganine (Figure 1). Subsequently, 3-ketosphinganine is converted to sphinganine (dihydrosphingosine), then to dihydroceramide, then to ceramide, which is considered the central molecule in the pathway of sphingolipid metabolism. Ceramide can be converted into several metabolites including: SM, sphingosine, ceramide 1-phosphate, glucosylceramide, and galactosylceramide. Sphingosine can be phosphorylated to sphingosine 1-phosphate (S1P) by sphingosine kinases (SKs) (isoforms 1 and 2). The majority of ceramides are generated by the *de novo* pathway on the endoplasmic reticulum; however, there is a “salvage” pathway that can generate ceramide via the breakdown of sphingolipids such as SM, predominantly by acid sphingomyelinase in the lysosome and also extracellularly in the circulation (12, 13) (Figure 1). In addition, ceramide can be broken down to sphingosine and regenerated creating a balance between the bioactive molecules S1P and ceramide. Generally, ceramide is thought to be pro-apoptotic and S1P are thought to be pro-survival (14–16).

Sphingolipid nomenclature is derived from the fatty acid attached, the number of the carbon atoms in the fatty acid and the number of saturated carbons in the fatty acid. A C16:0 sphingolipid denotes the presence of 16 carbon-long fatty acid chain attached to the sphingosine backbone, whereas a C18:0 and C24:0 sphingolipid denotes the presence of 18 and 24 carbon in the fatty acid side chain, respectively. A C16:2 sphingolipid includes a 16 carbon-long fatty acid, with 2 carbons that are unsaturated (two double bonds). Sphingosine and dihydrosphingosine contain two stereogenic centers at the sites of the 2-amino and 3-hydroxyl groups, thus giving rise to a total of eight isomers: d-erythro, l-threo, l-erythro and d-threo of sphingosines and dihydrosphingosines. Therefore, sphingosine (d18:1) is d-erythro-sphingosine, and dihydrosphingosine (d18:0) is d-erythro-dihydrosphingosine. S1P can be dephosphorylated to sphingosine by sphingosine phosphatase and can be irreversibly degraded by the enzyme sphingosine phosphate lyase resulting in the formation of hexadecenal and phosphoethanolamine (Figure 1). Phosphoethanolamine is an ethanolamine derivative that is used to construct two different categories of phospholipids: glycerophospholipids and sphingophospholipids (e.g., sphingomyelin).

Glycerophospholipids are a class of lipids that have a hydrophilic “head” containing a phosphate group, and two hydrophobic “tails” derived from fatty acids, joined by a glycerol moiety. The two fatty acids may be the same, or different, and are usually in the 1,2 positions (though they can be in the 1,3 positions). The phosphate group can be modified with simple organic molecules such as choline, ethanolamine or serine to generate phosphatidylcholine (PC), phosphatidylethanolamine (PE), or phosphatidylserine (PS), respectively. For example PE, also known as 1-palmitoyl-2-linoleoyl-GPE (16:0/18:2), consists of a combination of glycerol esterified with the two fatty acids, palmitate (16:0) and linoleate (18:2), and phosphoric acid.

Sphingolipids are typically measured using mass spectroscopy with a triple-quadrupole mass spectrometer alone or with high performance liquid chromatography in tandem with high performance liquid chromatography, which provides more sensitivity and specificity of the analyses. Analytical approaches are either non-targeted (shotgun) lipidomics or targeted lipidomics; both approaches have been adopted in plasma sphingolipidomics analysis in SLE (17, 18).

### Sphingolipids as Biomarkers of Disease

Sphingolipids can be found in plasma, urine, synovial fluid cerebrospinal fluid, and more recently biopsies, specifically kidney biopsies (19). Sphingolipids are found circulating in blood as part of the lipoprotein particles (VLDL, LDL, and HDL). The most studied sphingolipid in the circulation, S1P, originates mostly from red blood cells and platelets, and can be transported mainly on HDL particles and also bound to albumin (20–23). Sphingolipids have shown promise as potential biomarkers in diseases such as coronary artery disease (CAD), heart failure, several cancers, asthma, chronic obstructive pulmonary disease (COPD), Alzheimer’s disease, and several other diseases and disorders including autoimmune diseases such as rheumatoid arthritis and multiple sclerosis. Further information regarding sphingolipids as biomarkers of disease has been comprehensively reviewed in recent publications (20). In this article, we will review the specific advances in the potential consideration of sphingolipids as diagnostic and prognostic biomarkers of SLE and its comorbidities.

## Diagnosis of SLE

Being a complex and multifaceted disease, SLE is not fully understood. The most recent classification criteria for SLE are the 2019 European League Against Rheumatism/American College of Rheumatology (EULAR/ACR) showing an improved sensitivity of 96.1% and specificity of 93.4% over other diagnostic criteria (7). To satisfy diagnostic criteria, a patient must have at least one positive anti-nuclear antibody (ANA) screen, as well 10 or more points in additive weighted criteria (24). These include seven clinical involvement areas and three immunological domains (24). The clinical involvement considers constitutional, hematological, neuropsychiatric, musculocutaneous, serosal, musculoskeletal, and renal systems (24). The Immunological criteria include the presence of antiphospholipid antibodies (APLA), complement proteins and SLE-specific antibodies like anti-ds DNA, anti-ss DNA, anti-Smith (Sm), and anti-histone DNA (24). To meet EULAR/ACR criteria a patient must only exhibit diagnostic criteria once in their lifetime (24). The new guidelines are more sensitive and specific; however, diagnostic criteria require disease activity and immunologic dysfunction.

### Typical SLE Patient and Initial Evaluation

A typical SLE patient can present with a myriad of symptoms from any organ system. Usually patients present with fatigue, fever, myalgias, weight changes, arthralgia, and lupus nephritis (25, 26). Patients can also present with neuropsychiatric symptoms from lupus cerebritis or strokes. Less commonly SLE can cause myocardial infarctions, thromboembolic disease, or other vasculitides (27–29). Overall lupus is a very versatile and promethean disease that can have devastating impacts on any organ system.

### SLE and Clinical Lipidology

It is known that SLE patients are at increased risk for atherosclerosis and cardiovascular disease (CVD). Whereas it is rare for women without autoimmune disorders to have a myocardial infarction before the age of 55, young SLE women 35–44 years old, have 50-fold the incidence rate in comparison (30). Atherosclerosis and CVD are most commonly associated with lipid dysregulation so it is only fitting that in the evaluation of SLE, HDL, and LDL profiles are considered.

A high HDL cholesterol (>60 mg/dL), low LDL cholesterol (<100 mg/dL), and low total cholesterol (<200 mg/dL) are ideal for a healthy individual. HDL is known to be protective against atherosclerosis; however, this concept does not hold up in the context of SLE. Changes in HDL, LDL, and total cholesterol have been recently evaluated as potential biomarkers of SLE, specifically HDL levels and changes in its protective function (31). In a cross-sectional study, the lipid profile of 71 young (20–30 years old) women with SLE and age matched controls were studied with the goal of describing the relationship of lipids with disease severity (31). Triglycerides and VLDL cholesterol levels were significantly increased in the SLE group, while levels of total cholesterol, HDL cholesterol, LDL cholesterol, Apo A, and Apo B were significantly reduced. Disease severity correlated with the level of dyslipidemia. This allowed the authors to surmise that disease severity also correlated with the risk for atherosclerosis (31). In a study conducted by McMahon et al. (32), the functional ability of HDL to prevent the oxidation of LDL was determined in 154 SLE patients. Oxidation of LDL is a known early step in the development of atherosclerosis. They found that 44.7% of the SLE patient group had pro-inflammatory HDL in comparison to 4.1% of the healthy control group (32). Also, levels of oxidized LDL correlated with levels of pro-inflammatory HDL (*r* = 0.37, *p* < 0.001). SLE patients with CAD were found to have significantly higher pro-inflammatory HDL scores than patients without CAD (32). The SLE patients therefore were more likely to have HDL that was unable to prevent the formation of atherosclerosis. Changes in HDL functionality including lipid composition, increased oxidation, and impaired cholesterol efflux activity in SLE patients with the possibility of targeting these irregularities for treatment have been recently reviewed (33). Whereas pro-inflammatory HDL was shown to be a predictor of atherosclerosis; it does not account for the many deleterious effects of SLE on other organ systems; lupus nephritis still impacts a significant portion of SLE patients (8).

Because changes in HDL structure may account for the increased risk of CVD and atherosclerosis, sphingolipid composition of HDL may also be key in determining the level of dysregulation in HDL functionality. Among lipoproteins in the circulation, HDL is the major carrier of S1P, with about 60% of plasma S1P under normal healthy conditions (20–23). Compared to HDL2 particles, HDL3 particles are rich in S1P and low in SM, with the S1P concentration in HDL3 particles averaging twice the levels in HDL2 particles, and SM content is twofold less than in HDL2 particles (34, 35). Because HDL particles are heterogeneous with differing sizes, shapes, densities, protein compositions, and lipid diversity and in a constant state of remodeling and interconversion, this begs the question: could alteration of S1P distribution among lipoprotein particles and changes in S1P levels in HDL3 particles be pro-inflammatory and inhibitory of the normal anti-atherosclerotic activity of HDL? A closer look needs to be taken into the possible roles which sphingolipids in lipoproteins are playing in the pathogenesis of SLE and its comorbidities.

## SLE Activity and Damage Indices

With implications of sphingolipids in the SLE setting, extending into disease severity is a pertinent discussion. There are several different scales addressing disease activity and damage associated with SLE (36). The two main indices used to assess activity are the Systemic Lupus Activity Measurement (SLAM) and Systemic Lupus Erythematosus Disease Activity Index (SLEDAI) (37). Damage is also evaluated with the Systemic Lupus International Collaborating Clinics Damage Index (SLICC/ACR) scales. Kidney involvement is being assessed via the British Isles Lupus Assessment Group Scale (BILAG). To clarify the difference between activity and damage, activity represents symptoms caused by the disease, in this case SLE, at the time of the evaluation. Damage on the other hand is permanent, irreversible change caused by disease or drugs. Activity can fluctuate over the disease course; therefore, SLEDAI, SLAM and BILAG can go up and down over time. Since damage is irreversible, the damage score determined by SLICC/ACR can only increase or stay the same.

### Activity Indices

The SLAM originated in 1989 and has since been revised (SLAM-R). SLAM-R scale measures disease activity in the last month with clinical and laboratory manifestations weighted by severity. There are nine organ system considered and seven laboratory values, but it does not include immunology (36, 38). Each variable is scored from 0 to 3 with a maximum score of 81, with a score of 7 being clinically significant for treatment (39). SLAM considerers several objective measurements; however, it does not consider the patients opinion of the symptoms.

Systemic Lupus Erythematosus Disease Activity Index originated in 1992 and revised in 2002 (SLEDAI-2K) (40). This scale measures disease activity in the last 10 days and considers 24 weighted objects over nine organ symptoms and laboratory values including immunology. The range can go up as high as 105, with no activity being 0, mild 1–5, moderate 6–10, high 11–19, very high ≥20 (40). Since this scale considers objective rather than subjective information, it correlates less with patient perception of health and is less sensitive to change, although the test itself is considered to have high reliability (36, 39, 41).

British Isles Lupus Assessment Group Scale originated in 2004 and is different from all the other scales in that it considerers disease impact in each organ system that can be combined to give an overall assessment (37). This is also the only scale where progression over time is documented. Nine organs are considered; however, immunology is not. The index can be given as new, same, worse, or improving, which allows for greater sensitivity to change. This is the most comprehensive evaluation scale. Evaluations are reported as A-very active disease, B-patient needs an increase in treatment, C-stable or mild disease, D-previous organ involvement but no current activity, and E-no organ involvement and there has never been organ involvement. Thus, SLEDAI and BILAG are the two main activity indices; SLAM is rarely used and not included in any of the composite outcome measures used in clinical trials. All three activity indices are used to assess total disease activity but only BILAG assesses organ-specific activity.

### Damage Indices

Systemic Lupus International Collaborating Clinics Damage Index has been accepted by the American College of Rheumatology as an index to assess end organ damage as a result of SLE. Forty one items are covered over 12 systems based on points going up to 47 (38). Patients rarely go above 12 points, but this score has a prognostic value. Higher scores mean that there is more damage, which typically presents after recurrent flares or chronic disease, which has led to accumulation of damage. This index has value in giving the patient quality reports of how their SLE will affect their life going forward.

These are a few of the more widely accepted indices and scales for SLE-related organ damage. While these indices are considered reliable and valid, there is variations in each one that allow for different strengths and weaknesses. It would be ideal to have one test or set of test that can consider organ damage, overall disease involvement, and prognosis. Here we consider the possibility of sphingolipids to be added in addition to the current markers of SLE to form a more comprehensive disease index.

## Sphingolipids as Biomarkers for SLE and Its Comorbidities

Blood as a testing medium is very valuable as the medical field has countless tests that rely on serum and plasma for clinical indications. Yet, there is still more to be explored as far as metabolites that can be useful in the diagnosis of diseases (42). In 2011, the nuclear magnetic resonance technology was used to establish a metabolic profile for lupus patients, and showed a clear distinction from profiles of healthy controls and patients with rheumatoid arthritis (43). Tested metabolites such as amino acids, glycoproteins, TCA cycle intermediates, and lipids showed a sensitivity of 60.9% and specificity of 97.1% in predicting the diagnosis of SLE (43).

More recently, with the extensive role of sphingolipidomics being identified (23), Li et al. (44) evaluated serum samples from 32 SLE patients (17 with active and 15 with inactive disease) and 32 healthy controls using untargeted lipidomics as well as metabolomics. Groups, which consisted of patients with SLE flare, were defined as active, and those, in which the disease was quiescent were defined as inactive. Throughout either phase the disease was considered to be in a remitting relapsing cycle. In comparison to the healthy individuals, SLE patients differentially regulated 16 lipids, nine of which were up regulated and seven were down regulated (44). The sphingolipids: C42:2 SM and several ceramides including C18:0 ceramide, C16:0 ceramide with a sphinganine backbone (d18:0), and C24:2 ceramide with a sphingadiene backbone (d18:2) were significantly elevated (*p* = 0.02587, *p* = 0.00626, *p* = 0.000545, and *p* = 0.004681, respectively) in the SLE patients (44). Acylcarnitines 7:0, 8:2, 9:0, 10:0, 22:5, and 22:6 were significantly down regulated (*p* = 0.023883, *p* = 0.023883, *p* = 0.034984, *p* = 0.02626, *p* = 0.013191, and *p* = 0.012735, respectively); however, PE 34:2, diacylglycerol 36:4, ether phosphatidylcholine 26:0, ether phosphatidylcholine 36:1, and arachidonic acid 20:4 were increased (*p* = 0.010404, *p* = 0.023883, *p* = 0.02626, *p* = 0.02626, and *p* = 0.013191, respectively) in the SLE patients (44).

In another study aimed to determine the lipid profile of SLE patients, the serum samples from 30 SLE patients and 30 controls were analyzed using shotgun (untargeted) mass spectrometry (17). Lu et al. (17) showed a significant change in the sphingolipid profile of SLE patients. They found that overall ceramide levels did not change; however, the levels of C22:0 and C23:0 ceramide species decreased, and the levels of C24:0 ceramide increased (*p* < 0.001, *p* < 0.05, *p* < 0.05, respectively) (17). In addition, diacyl phosphatidylethanolamine (dPE) 16:0/18:2, 18:0/18:2, 16:0/22:6, 18:0/20:4, 18:0/22:6, and lysoPC 18:2 were significantly altered (*p* < 0.05) in comparison to the controls. Although the total SM content did not change, the levels of SM species comprised of C18 acyl chains significantly increased in SLE patients when compared to controls (17). This differential regulation of sphingolipids shows that lipidomics with a focus on sphingolipidomics have the potential to be used as a screening tool for early diagnosis of SLE.

### Sphingolipids and SLE Disease Activity

The impact of serum lipidomics was shown to extend into disease activity of SLE. Lu et al. (17) found a significant positive correlation between SLEDAI, a currently used disease activity index, and ethanolamine plasmalogen (pPE) 18:0/18:2 (*p* = 0.031). In addition there was a positive correlation between IL-10 and pPE 18:0/18:2 (*p* < 0.0001). Although IL-10 is considered an anti-inflammatory cytokine, in SLE its function is counterintuitive. Whereas IL-10 increases the survival, proliferation and differentiation of B cells, so that antibodies are produced to fight an infection; when there are autoreactive B cells such as in the case of SLE, the formation of autoreactive antibodies increases (45, 46). Lu et al. (17) showed that sphingolipids could provide additional biomarkers for disease activity in SLE and warrant further exploration.

In a cross sectional study, Checa et al. (41) investigated the association between clinically significant systemic disease activity and renal disease activity with circulating sphingolipids in SLE patients. They measured 27 sphingolipids in serum samples of 107 female patients with SLE and 23 healthy controls and compared the values against the two commonly used SLE disease activity indices: SLAM and SLEDAI (41). Damage was assessed with the SLICC and renal activity was accessed with BILAG (41). The results showed a significant increase in several ceramide species including C16:0, C18:0, C20:0, and C24:1 (*p* < 0.01, *p* < 0.01, *p* < 0.05, *p* < 0.05, respectively), hexosylceramides (Hex-Cer) C16:0, C18:0, C18:1, and C24:1 (*p* < 0.001, *p* < 0.01, *p* < 0.05, *p* < 0.001, respectively), SM C24:1 (*p* < 0.05), and dihydroceramide C16:0 (*p* < 0.05). There was a significant decrease in sphingosine (*p* < 0.05) and S1P (*p* < 0.01) in SLE patients when compared to the controls (41). In this same study, patients with SLE were also grouped according to their current disease activity with patients with a SLAM ≥ 7 and SLEDAI ≥ 6 being classified as having active disease. End organ damage was considered to be SLICC ≥ 2. SLE patients with SLAM ≥ 7, SLEDAI ≥ 6, and SLICC ≥ 2 were compared to SLE controls that did not meet the disease activity or damage qualifications (41). For SLAM and SLEDAI activity groups, result showed that C24:1 ceramide (*p* < 0.01, *p* < 0.01), C16:0 Hex-Cer (*p* < 0.01, *p* < 0.001), C24:1 Hex-Cer (*p* < 0.01, *p* < 0.01), C16:0 ceramide/S1P ratio (*p* < 0.001, *p* < 0.001), and C24:1 ceramide/S1P ratio (*p* < 0.001, *p* < 0.001) were elevated, respectively, when compared to controls. Result also showed that C24:1 ceramide (*p* < 0.01), C16:0 Hex-Cer (*p* < 0.01), C24:1 Hex-Cer (*p* < 0.01), C16:0 ceramide/S1P ratio (*p* < 0.01), and C24:1 ceramide/S1P ratio (*p* < 0.001) were elevated in SLICC damage groups when compared to controls. In addition, cystatin C was significantly increased in SLAM ≥ 7, SLEDAI ≥ 6, and SLICC ≥ 2 groups (*p* < 0.001, *p* < 0.01, *p* < 0.001, respectively) when compared to controls (41).

The ratios C16:0 ceramide/S1P and C24:1 ceramide/S1P were shown to be correlated with SLAM and SLEDAI and were best discriminators of ongoing disease, but were not a useful discriminator of organ damage (41). Checa et al. (41) showed significant differences in C16:0 ceramide and C16:0 ceramide/S1P ratio between SLAM < 7 and SLAM ≥ 7 groups (*p* < 0.05, *p* < 0.01, respectively), with higher levels correlating with higher SLAM values (41). This also was the case for SLEDAI. C16:0 ceramide and C16:0 ceramide/S1P ratio showed a significant difference between SLEDAI < 6 and SLEDAI ≥ 6 groups (*p* < 0.05, *p* < 0.001, respectively), with higher levels correlating with higher SLEDAI values (41). Hex-Cer C16:0 and C24:1 levels were the only species with significant difference between current and no prior or inactive renal involvement, when compared to BILAG, with levels increased in SLE patients that were currently experiencing renal involvement (*p* < 0.05, *p* < 0.05, respectively) (41).

Checa et al. (41) also showed that following immunosuppressive treatment, sphingolipids were normalized to the levels seen in the healthy controls further supporting the use of sphingolipids as indices of SLE disease activity. This also opens the door for the consideration of the use of sphingolipids in monitoring the progress of treatment with normalization of sphingolipid levels possibly being used as a cutoff point.

### Sphingolipids and SLE Prognosis and Sphingolipid Response to Treatment

Rituximab is a human monoclonal antibody B cell-targeting therapy that is used to treat autoimmune diseases and certain cancers. In a study with the focus of determining the effect of Rituximab on circulating plasma sphingolipids, sphingolipid measurements were performed before and after treatment with Rituximab (47). Ten SLE patients were screened for 34 sphingolipids before and after Rituximab treatment (47). Sphingolipids were down regulated following treatment and a course of disease improvement (48). C16:0 dihydroceramide and C16:0 glucosylceramide were shown to be significantly down regulated (*p* = 0.04 and *p* = 0.006, respectively), in addition to seven other sphingolipids that were shown to be down regulated using paired analyses. These results are in agreement with the study by Checa et al. (41), which compared sphingolipid levels before and 9.5±3.3 months after immunosuppressive treatment. Their results showed a decrease in the ceramide species C16:0, C18:0, C22:0, and C24:1 (*p* < 0.01, *p* < 0.05, *p* < 0.05, and *p* < 0.05, respectively), a decrease in the Hex-Cer species C16:0, C18:0, and C24:1 (*p* < 0.001, *p* < 0.01, and *p* < 0.01, respectively), a decrease in the SM C16:0 (*p* < 0.01) and in dihydroceramide C16:0 (*p* < 0.05) (41). S1P was significantly increased (*p* < 0.05) and C16:0 ceramide/S1P and C24:1ceramide/S1P ratios significantly decreased (*p* < 0.01, *p* < 0.01, respectively) following treatment (41). Both studies, which examined sphingolipid levels before and after treatment support the use of sphingolipid measurements as an evaluation of SLE status and as potential therapeutic biomarkers of response to treatment (41, 47).

### Sphingolipids and SLE Comorbidities

#### Kidney

One of the most common manifestations of SLE is lupus nephritis, with renal impairment occurring in 30–60% of adults and 70% of children with SLE (49). Although lupus nephritis is part of a larger systemic disease, it does not necessarily respond the same way to treatment. Renal impairment does not always improve with immune suppression or overall disease remission; however, progression to chronic kidney disease is not inevitable (49). Therefore, there is a need for organ specific evaluations and treatment apart from the typically considered immunosuppression SLE therapies. Li et al. (50) used high resolution mass spectrometry and liquid chromatography to characterize the metabolic profile of lupus nephritis patients. Serum samples from 32 lupus nephritis patients, 30 idiopathic nephrotic syndrome patients and 28 healthy controls were analyzed. Of the 14 potential biomarkers screened, sphingosine showed a significant decrease (*p* < 0.05) in lupus nephritis patients in comparison to healthy controls, with a sensitivity of 87.50% and specificity of 32.14% for identifying lupus nephritis (50). In the study by Checa et al. (41), levels of serum C16:0 Hex-Cer and C24:1 Hex-Cer were found to be increased (*p* < 0.01 and *p* < 0.05, respectively) with active renal disease when compared to BILAG.

Nowling et al. (51) investigated the role of glycosphingolipid metabolism in lupus nephritis. Glycosphingolipids are abundant in the kidneys, playing a role in inflammation, proliferation, and cellular regulation and abnormal glycosphingolipid metabolism have been implicated in several kidney and autoimmune diseases (52). Even after normalizing to creatinine and eGFR, C16:0 lactosylceramide (Lact-Cer) was found to be significantly elevated (*p* < 0.001) in urine of lupus nephritis kidneys when compared to healthy controls, suggesting that kidney dysregulation of glycosphingolipid metabolism may be involved in the pathogenesis of lupus nephritis (51). In addition, when glomeruli were probed for Lact-Cer, a low level of staining was detected in the control kidney biopsies but there was intense staining inside and outside of the glomeruli in biopsies from lupus nephritis patients (51). Nowling et al. (51) concluded that elevated levels of Lact-Cer in combination with elevated neuraminidases, the enzymes that generate Lact-Cer, suggest that renal dysfunction rather than systemic dysfunction is the cause of elevated Lact-Cer. As SLE can manifest in almost any organ system, and the organ involvement does not always follow the trend of the systemic disease, determination of urine Lact-Cer levels may be useful as an early biomarker for lupus nephritis, particularly when a systemic lupus flare is not apparent. Whereas routine clinical assessment plus ESR, anti-dsDNA and C3 tests can identify SLE flare, relapse of lupus nephritis can be more difficult to assess. This would require multiple renal biopsies, which is an invasive test that may not be justifiable given the risks. Urinary or plasma sphingolipids could prove to be a useful tool in predicting or identifying a lupus nephritis flare.

In a cross-sectional study, 82 patients were divided into three groups: healthy controls, SLE without renal injury and SLE with renal injury (lupus nephritis) based on their eGFR, and kidney biopsy results (53). Lupus nephritis patients were further stratified by severity of renal impairment. Sphingolipid levels were evaluated in the plasma and serum of venous blood drawn from the subjects. The results showed that C16:0, C18:0, C20:0, and C24:1 ceramides were significantly elevated in the serum of lupus nephritis patients when compared to healthy controls (*p* < 0.001, *p* < 0.001, *p* < 0.001, and *p* < 0.001, respectively), and to SLE patients without renal impairment (*p* < 0.01, *p* < 0.001, *p* < 0.001, and *p* < 0.001, respectively). Since circulating ceramides are carried on lipoproteins, plasma C16:0, C18:0, C20:0, and C24:1 ceramides were, as expected, also significantly elevated in lupus nephritis patients when compared to the healthy controls as well as SLE patients without renal impairment (53). Plasma dihydroceramide C24:1 was also elevated (*p* < 0.05) in lupus nephritis patients in comparison to SLE patients, suggesting that C24:1 dihydroceramide, and C16:0, C18:0, C20:0, and C24:1 ceramides could be useful in differentiating SLE patients with renal damage from those without (53). Sphingosine levels were significantly increased in the serum and plasma of lupus nephritis patients (*p* < 0.01, *p* < 0.001, respectively) when compared to healthy controls. Li et al. (50) showed that serum sphingosine was significantly different between lupus nephritis patients and healthy controls; however, in their study sphingosine levels were decreased rather than increased.

Patyna et al. (53) found that in serum, sphinganine (dihydrosphingosine) was significantly elevated in lupus nephritis patients when compared to SLE and healthy controls (*p* < 0.05, *p* < 0.05, respectively). In plasma, S1P and sphinganine 1-phosphate [dihydrosphingosine 1-phosphate (dhS1P)] were found to be elevated in all SLE patients, with or without lupus nephritis, in comparison to healthy controls (*p* < 0.05, *p* < 0.05, respectively). Ceramide C24:1 showed the most potential of being used as a biomarker of lupus nephritis as it remained strongly elevated in lupus nephritis patients (*p* = 0.0001), even when compared to SLE patients without kidney disease (53). These data show the potential value of assessing sphingolipid changes in lupus nephritis and supports the use of sphingolipidomics as a tool to pinpoint a biomarker(s) for early identification of lupus nephritis in SLE patients.

#### Cardiovascular System

Systemic lupus erythematosus is associated with premature atherosclerosis and accelerated CVD. A combination of different factors including but not limited to endothelial dysfunction, genetic markers, diminished endothelial nitric oxide production, proinflammatory neutrophils, dysregulated T cells, dyslipidemia, autoantibodies, and immune complexes, contribute to the development of CVD in SLE patients (54). This creates a stark difference of atherosclerosis between patients with SLE and healthy controls even when similar Framingham risk factors are shared (54). A prospective study with a 5-year follow-up showed that 32% of SLE patients had atherosclerosis, whereas 4% of the healthy controls developed atherosclerosis (55). Neutrophils have been implicated as mediators of vascular damage with proinflammatory neutrophils promoting endothelial leakage through degradation of the extracellular matrix components, which in turn allows for endothelial dysfunction (56).

A hallmark of SLE is the formation of autoantibodies and immune complexes, which can damage the vascular endothelium creating a proinflammatory state (54). In SLE, anti oxLDL IgG antibodies are significantly elevated (54). Oxidized LDL are at increased levels in women with SLE, and anti-oxidized phospholipid antibodies showed that some APLA target oxidized LDL (57). The role of APLA has been investigated in SLE patients in regards to its connection to valvular heart abnormalities. Ruiz et al. (58) selected 70 SLE patients based on their antiphospholipid antibody levels as well as the presence or absence of regurgitation, artificial valves, stenosis, thickening and Libman–Sacks endocarditis. They found a correlation between abnormality in any valve and antiphospholipid antibody levels greater than 20 units/mL (*p* = 0.035); however, significance varied when antiphospholipid antibody levels were compared to individual valvular lesions. The authors concluded that high levels of APLA, especially ≥40 units/mL, are significantly associated with heart valve disease (58).

It is well established that macrophages play a major role in the formation of oxidized LDL and the development of atherosclerosis. Under oxidative stress and inflammatory conditions, released cytokines, growth factors, and bioactive mediators recruit monocytes to the vessel wall and cause the monocytes to differentiate into resident macrophages. Macrophages endocytose oxidized LDL and oxidized LDL-containing immune complexes and become lipid-laden cells known as foam cells (59, 60). Foam cells harden and release cytokines and other mediators that contribute to the formation of atherosclerosis. Because SLE is an autoimmune disease that results in a chronic, remitting and relapsing, pro-inflammatory state, several studies investigated the role of monocytes/macrophages in accelerated atherosclerosis in SLE (59).

Sphingolipids, particularly S1P has been implicated in several diseases including but not limited to atherosclerosis, multiple sclerosis, and other autoimmune disease (61–63). In a study involved 308 patients in need of coronary angiography, increased serum S1P was found to be a stronger predictor of CAD (*p* < 0.001) than the traditionally considered risk factors including gender, age, family history, lipid profile, hypertension, and smoking (62). A score that included S1P levels, gender and age showed a strong relationship with CAD severity (*p* < 0.01) (62). Because SLE patients do not follow the normal age and gender pattern for atherosclerosis and CVD, but instead follow an accelerated disease course, the traditional risk trends do not accurately assess their risk. This added identification marker of S1P may be what is needed to more accurately prognosticate an SLE patient cardiovascular risk prior to an adverse health event. Mechanistically, S1P was shown to induce the release of inflammatory mediators TNF-alpha, cyclooxygenase and prostaglandins in macrophages (64). Therefore, serum/plasma S1P levels in combination with other risk factors could be advantageous in identifying risk for CAD in SLE. This begs the question of whether targeted treatment that down regulates the formation of S1P and/or blocks its receptors would be helpful in the prevention of atherosclerosis.

African-Americans are three times more likely than Whites to have lupus and develop severe symptoms including accelerated CVD (65). Furthermore, African-Americans normally have lower triglycerides and higher HDL cholesterol levels than other ethnicities; however, paradoxically they have increased risk of CVD (66). Our group has recently examined the influence of race on plasma sphingolipid profiles in SLE patients and associations of sphingolipid levels with comorbid atherosclerosis and SLE disease activity (18). Compared to healthy Whites, healthy African-Americans had higher SM levels and lower Lact-Cer levels. However, irrespective of race, SLE patients had higher levels of ceramides, and sphingoid bases (sphingosine and dihydrosphingosine) and their phosphates compared to healthy subjects. Within African-American subjects, SLE patients had higher levels of ceramides, Hex-Cer, sphingosine, and dhS1P compared to African-American controls. Within White subjects, SLE patients exhibited higher levels of sphingoid bases and their phosphates, but lower ratios of C16:0 ceramide/S1P and C24:1 ceramide/S1P compared to White controls. Within White SLE patients, those with atherosclerosis exhibited lower levels of sphingoid bases compared to those without. In contrast, within African-American SLE patients, those with atherosclerosis had higher levels of sphingoid bases and SMs compared to those without. Comparing White SLE patients with atherosclerosis with African-American SLE patients with atherosclerosis, the latter had higher levels of certain sphingolipids. Notably, C16:0 ceramide/S1P ratio in SLE patients, and levels of C18:1 and C26:0 Lact-Cer, C20:1 Hex-Cer, and sphingoid bases in SLE patients with atherosclerosis could be dependent on race and indicate that there are race-dependent factors, which may regulate the homeostasis of the sphingolipid metabolic and signaling pathways, including the activity of sphingolipid metabolizing enzymes, which may influence the levels of circulating sphingolipids. As for disease activity, plasma levels of sphingosine, C16:0 ceramide/S1P ratio and C24:1 ceramide/S1P ratio significantly correlated with SLEDAI in the African-American but not White SLE patients (18). Further ethnic studies in SLE cohorts are needed to assess the use of plasma sphingolipidomics as an added diagnostic tool.

Other than the probable race effect, Hammad et al. (18) study excluded the nephritis comorbidity, which was included in the European Checa et al. (41) study. In the setting of lupus nephritis, it is possible that in the Checa et al. (41) study the plasma S1P fraction bound to albumin (22) is depleted due to its excretion with the urine (albuminuria). Patyna et al. (53) showed that S1P and dhS1P levels were higher in plasma samples of SLE patients (irrespective of renal function) compared to healthy controls, which is in agreement with data from the Hammad et al. (18) study. In the Patyna et al. (53) study sphingosine and C16:0, C18:0, C20:0, and C24:1 ceramide levels were elevated only in SLE patients suffering from impaired renal function, compared to healthy controls and SLE patients without impaired renal function. Urinary loss of S1P due to lupus nephritis could inflate the C24:1 ceramide/S1P ratio possibly causing differences in correlations with SLEDAI. The lack of notable correlations between SLEDAI and plasma sphingolipid levels among Whites in the Hammad et al. (18) study compared to the Checa et al. (41) study could be due to several confounding factors such as the fact that Whites with SLE tended to be healthier, sphingolipids may not be associated with disease severity among Whites, and/or the effect of the small sample size. The inclusion of detailed information about pre-analytical and analytical confounders in clinical studies are particularly essential in assessing the reliability of a potential biomarker.

Sphingolipids were also recently evaluated via targeted lipidomics to determine if sphingolipid levels would be a valuable cholesterol-independent biomarker for CAD (67). Poss et al. (67) compared the serum sphingolipid profile in 462 individuals with familial CAD and 212 population based controls. When compared to the control group, 30 of the 32 sphingolipids tested were significantly elevated in the CVD group. Based on their results. Poss et al. (67) formed a sphingolipids inclusive CAD risk score which they abbreviated as SIC. This score included the sphingolipids: dihydroceramide C18:0 (*p* = 2 × 10^–16^), ceramides C18:0, C22:0, and C24:0 (*p* = 5.40 × 10^–16^, *p* = 3.63 × 10^–11^, *p* = 1.61 × 10^–15^, respectively), dihydro-SM C24:1 (*p* = 1.40 × 10^–10^), SMs C18:0, C24:0 (*p* = 2.54 × 10^–6^, *p* = 1.44 × 10^–9^, respectively), and sphingosine (*p* = 2 × 10^–16^) (67). SIC had better discriminatory power for CVD than the widely accepted LDL-cholesterol levels showing C-statistics of 0.79 and 0.69, respectively (67). The significance of SIC as a predictor of CVD independent of LDL-cholesterol suggests that circulating sphingolipids in addition to the current risk factors could be used to more accurately assess an SLE patient’s risk of CVD.

#### Brain

One other major complication of SLE is neuroinflammation, which is also known by neuropsychiatric SLE. Normally, the blood brain barrier in conjunction with chemical mediators, separate the central nervous system (CNS) from foreign substance and chemical mediators in the systemic circulation, tightly controlling the nervous system. When damage occurs in the neural parenchyma, cytokines, and chemokines that activate glial cell (neuron support cells) are released and result in the release of more pro-inflammatory mediators. Prolonged glial cell activation (gliosis) can cause scaring, cell death of the surrounding CNS cells and damage to the protective blood brain barrier. Damage in the blood brain barrier allows for lymphocytes and other inflammatory cells to enter the brain, further complicating the inflammatory process. Sphingolipids are abundant in the brain and CNS in general. In addition, studies have shown that sphingolipids play a significant role in the pathogenesis of several neuroinflammatory disorders such as Alzheimer’s disease and Parkinson’s disease (68, 69). During early phases of neuroinflammation, astrocytic sphingolipid alterations, which include an increase in ceramide and decrease in SKs that generate S1P were reported (68, 70). More information regarding the role of sphingolipids in neuroinflammation were reviewed previously (68, 69, 71).

The most common neurological manifestations found in SLE are cognitive dysfunction, headache and mood disorders, with no good serological markers for this SLE complication. Gangliosides are a family of sialylated glycosphingolipids expressed in the outer leaflet of the plasma membrane and they are abundant in the nervous system, particularly at synapses, and involved in neurotransmission at the neuromuscular junction. Gangliosides have a hydrophilic sugar chain that contains antigenic determinants and a hydrophobic ceramide. In humans, gangliosides elicit a T-cell-independent IgM response (72), which can cause leakage of the blood brain barrier or bind to neuronal gangliosides to create a neuromuscular block as in multiple sclerosis (73). A number of studies explored the presence of anti-ganglioside antibodies in SLE patients with neuropsychiatric manifestations and peripheral neuropathy and reported contradictory results (74).

## SLE and Sphingolipids in Experimental Animals

Sphingolipids and their role in the pathogenesis of SLE have been investigated in animal studies. Because B lymphocyte and macrophages/dendritic cells were found to contribute to SLE pathogenesis through toll-like receptor (TLR)-stimulated cytokine production (59, 75), TLR signaling was induced to examine changes in sphingolipid metabolism in an SLE-prone mouse model (76). TLR induction caused abnormal expression of several key enzymes in sphingolipid metabolism including, SM phosphodiesterase 3, sphingosine 1-phosphate phosphatase 2, ceramide kinase and UDP glycosyltransferase 8 in B cells and macrophages (76). SM phosphodiesterase, UDP glycosyltransferase 8 and ceramide kinase were decreased in splenic B cells (*p* < 0.001, *p* < 0.01, and *p* < 0.001, respectively). On the contrary, B cell sphingosine 1-phosphate phosphatase 2 was upregulated in SLE-prone mice (*p* < 0.01). In macrophages of SLE-prone mice, SM phosphodiesterase and UDP glycosyltransferase 8 were decreased (*p* < 0.05 and *p* < 0.01, respectively) (76). In macrophages and B cells from SLE patients and SLE-prone mice, dysregulated sphingolipid metabolism enhanced the proinflammatory response by prolonging survival and causing an excess immunological response to TLR signaling (76). Sphingolipid metabolism could therefore have a potential to be used as a target to modulate autoimmune response.

In addition to macrophages and B cells, dendritic cells have also been implicated in the pathogenesis of SLE. Dendritic cells are antigen-presenting cells that activate other immune cells such as T cells in the induction of an inflammatory response (59, 75). A specific type of dendritic cell, plasmacytoid dendritic cell (pDC), secrete large quantities of interferon (IFN). SLE is an autoimmune disease characterized by overproduction of type 1 IFN (IFN-1) (77, 78). Recently, Mohammed et al. (79) examined the role of sphingosine kinase 2 (SK2) in the pathogenesis of SLE in the pristane-induced murine lupus mode. Pristane (common name for tetramethylpentadecane)-induced lupus is a murine model of systemic SLE that is suited for examining links between dysregulated IFN-I production and the pathogenesis of human SLE, which is also associated with high levels of IFN-I (77, 78, 80). SK2 as well as the isoform SK1 phosphorylate sphingosine into the bioactive molecule S1P. When comparing SK2 knockout mice with wild-type mice, dendritic cell markers were found to be upregulated in SK2 knockout mice, suggesting that SK2 is an endogenous negative regulator of pDCs (79). The effects of SK2 knockout were also evaluated in the pristine-induced lupus mice model. When comparing SK2 knockout mice and wild-type mice injected with pristane, SK2 knockout significantly increased the percentage of pDC cells (*p* < 0.001). Nevertheless, the onset of lupus symptoms in the SK2 knockout pristane-induced lupus mice was found to be unaffected and there were no noticeable effects on the IFN signature that is typical of lupus, when compared to SK2 knockout mice with no pristine induction (79). Since clinical lupus symptoms were not improved by SK2 knockout in the pristine-induced lupus mice, it might not be recommended as a method for treatment; however, further investigation into this process is warranted to investigate factors that could have masked the effects of SK2 knockout. S1P and dhS1P were significantly elevated (*p* < 0.01 and *p* < 0.05, respectively) in the SK2 knockout mice (79), which is interesting given that SK2 phosphorylates sphingosine to generate S1P. This suggests that there is more to this process that we do not understand. One possibility is that SK1, which also phosphorylates sphingosine and to a lesser degree dhS1P, is possibly being upregulated when SK2 is removed, allowing for the increase in S1P.

### Lupus Nephritis

In a study that specifically examined the inhibition of SK2 in the MLR/lpr model of lupus nephritis, serum and kidney tissue were evaluated for S1P and dhS1P (81). The data showed that dhS1P, which is phosphorylated by SK2 was significantly elevated in the serum and kidney tissue of the murine model of lupus nephritis (*p* = 0.012 and *p* = 0.0097, respectively). Since SK2 has higher affinity for dihydrosphingosine than SK1, an SK2 inhibitor, ABC29640, was used on MLR/lpr mice to determine its effects on lupus nephritis. The SK2 inhibitor ABC29640-treated mice showed improvement in renal pathology of lupus nephritis; however, the classic markers of dsDNA, albuminuria and IgG deposition did not show improvement (81). In addition, this treatment decreased serum S1P (*p* < 0.05) and dhS1P (*p* < 0.01), but there was an increase in renal tissue dhS1P (*p* < 0.001) (81). Snider et al. (81) suggested that the therapy works downstream of immune complex formation and deposition into the kidney tissue. Similar to the study by Mohammed et al. (79), there was a question as to whether inhibition of SK1 could prevent the accumulation of dhS1P in kidney tissue and therefore improve glomerular pathology, albuminuria, and kidney function (81). One finding that was in common in both studies is that serum S1P and dhS1P where increased in the setting of lupus nephritis in the pristane-induced mice similar to that observed in the MRL-Lpr lupus mice (79, 81).

The role of S1P and its receptors in the production of proinflammatory cytokines as well as leukocyte trafficking has been established (71, 82, 83). In fact, FTY720 (fingolimod), an immunomodulatory drug which targets the S1P receptor (84), was approved for treatment of multiple sclerosis (85) and has been investigated as a treatment for lupus nephritis in experimental animals (86–89). In mouse models, FTY720 has been shown to increase survival as well as prevent end-stage glomerular disease via decreasing lymphocyte trafficking and increases their sequestration in lymph nodes (82, 86).

Nowling et al. (51) evaluated the role of glycosphingolipid metabolism in lupus nephritis in humans as well as in mice. Glucosylceramides and Lact-Cer levels were significantly elevated in the urine of lupus nephritis mice in comparison to nonnephritic lupus mice and healthy controls (*p* < 0.001 and *p* < 0.001, respectively) (51). Notably, in urine of lupus nephritis mice, Lact-Cer levels were significantly elevated prior to proteinuria (51), which is one of the earliest tests for kidney damage in lupus nephritis in humans (*p* < 0.001). This supports the idea that glycosphingolipids and/or other sphingolipids could be biomarkers for early detection of lupus nephritis with their metabolism possibly being amendable to therapy. While the full pathogenesis of lupus nephritis remains unclear, there have been strides made in understanding the mechanisms that may prove helpful in future investigations. These lupus nephritis-specific findings could provide more insight into why mouse models have shown that progression of lupus nephritis to chronic kidney disease is not inevitable even when SLE persists (49). There are several disease checkpoints that have been determined to be amenable to therapy.

### Vascular Disease

Systemic lupus erythematosus patients have an accelerated rate of atherosclerosis and CVD. A possible mechanism regarding the role of nitric oxide synthases in the regulation of endothelial function and atherosclerosis in SLE was investigated by our group (90). SLE patients have impaired endothelial nitric oxide synthase (eNOS) activity that may be overly compensated by inducible nitric oxide synthase (iNOS), which could result in inflammation. On the other hand, nitric oxide is a necessary metabolite for endothelium vasodilation and cardiovascular homeostasis in addition to playing a role in sphingolipid metabolism (90–94). To determine whether the lack of nitric oxide synthase impacts sphingolipid metabolism and atherosclerosis, lupus-prone MRL/lpr mice with *NOS2* (eNOS) deletion and MRL/lpr mice with *NOS3* (iNOS) deletion were used to determine changes in sphingolipid levels and immune complex deposition in the aorta, compared to their relative wild types (90). Mice with *NOS2* or *NOS3* gene deletions had significantly different sphingolipid levels, with plasma ceramides increasing 45 and 21%, respectively, when compared to the MRL/lpr control mice (*p* < 0.05) (90). The C22:0 and C24:0 ceramide species were increased in both *NOS2* and *NOS3* knockout mice compared to their counterpart control (*p* < 0.05). C24:1 ceramide was twofold higher in the *NOS2* knockout mice compared to their control mice, whereas there was no change in C24:1 ceramide in the *NOS3* knockout mice. In addition, S1P was significantly increased (21%) in both *NOS2* and *NOS3* knockout mice when compared to the MRL/lpr control mice (*p* < 0.05) (90). Gross examination of aortae from *NOS2* and *NOS3* knockout mice showed significantly higher lipid deposition scores compared to those from wild type controls (*p* < 0.05). Notably, nodule-like lesions in the adventitia were found in aortas from both *NOS2* and *NOS3* KO MRL/lpr mice. Immunohistochemical evaluation of the lesions revealed lipid-laden macrophages (foam cells), elevated SK1 expression, and deposition of oxidized low-density lipoprotein immune complexes in addition to the activated endothelium.

Since nitric oxide was found to inhibit ceramidases and therefore could lead to the increase of ceramide, the knockout of nitric oxide synthase would allow for uninhibited ceramidase activity (95–97). This in turn would provide a preponderance of the sphingosine substrate for SK1 to produce S1P. The functions of S1P has been extensively explored and has been found to be involved in the regulation of vascular permeability and inflammation allowing for leukocytes trafficking and cellular recruitment in a receptor-dependent manner (90, 98–101). While there are many steps in the pathogenesis of atherosclerosis, modulation of the metabolism of sphingolipids, including S1P, could improve the downstream effects of atherosclerosis seen in SLE patients.

## Conclusion and Potential Therapeutics

Targeting sphingolipids and their metabolism is beginning to be explored as a method of treating SLE and some of its complications. FTY720, a novel immunosuppressant with a structure resembling sphingosine and targets the S1P receptor, was used to determine if it had therapeutic potential in SLE (86, 89). FTY720 (2-amino-2-(2-[4-octyl-phenyl]ethyl)-1,3-propanediol hydrochloride) is a synthetic analog of a natural product that comes from the ascomycete *Isaria sinclairii* (102). FTY720 (2 μM) was administered to MRL/lpr mice from 4 months of age and were compared to control wild-type MRL/+ mice. Results showed apoptosis in >70% of CD4-negative/CD8-negative T cells in the spleen and lymph nodes, significantly decreased anti ds-DNA antibodies (*p* < 0.05), reduced deposition of IgG in kidney tissue, and increased survival (*p* < 0.01) in FTY720-treated MRL/lpr mice compared to the control group (89). At 9 months, 86.9% of the FTY720-treated group and 33.0% of the control group survived, suggesting that FTY720 suppressed autoimmunity and could possibly be investigated in humans as an adjunct therapy (89). In another study, the efficacy of FTY720 was evaluated in the prevention of end-stage renal disease in the BXSB mice (86). Similar to the study by Snider et al. (81), which explored the inhibition of SphK2 for treating lupus nephritis, an increase in survival (*p* < 0.05) was observed; however, a decrease in anti-DNA autoantibodies and deposition of IgG was not observed (86). Even with IgG deposition present in glomeruli, kidney tissue from the FTY720-treated mice still showed prevention of end-stage renal disease and was associated with normal-sized kidneys, decreased proteinuria (*p* < 0.0005) and no mesangial proliferation (86).

Previously Nowling et al. (51) observed elevated Lact-Cer and Hex-Cer levels in lupus nephritis MRL/lpr mice and suggested that these elevated glycosphingolipid levels could be caused by increased ganglioside GM3 catabolism, in part, by sterol regulatory element binding protein (SREBP)-1c induction of Neu1 expression, which breaks down gangliosides to generate Lact-Cer. Recently, they treated MRL/lpr mice with the neuraminidase inhibitor oseltamivir phosphate, in an effort to decrease renal GM3 and improve lupus nephritis in the mice (103). There was no significant difference in albuminuria and the scores of renal pathology (glomerular inflammation, proliferation, crescent formation, necrosis, and interstitial inflammation) between the oseltamivir phosphate-treated and vehicle-treated nephritic MRL/lpr mice. There was also no significant improvement of SLE in the oseltamivir phosphate-treated nephritic MRL/lpr mice in comparison to vehicle-treated mice, as measured by percentage of activated T cells, serum IgG levels, and splenomegaly (103). The authors suggested that accumulation of renal GM3 may be due to dysregulation of one or more of the glycosphingolipid ganglioside pathways. Therefore, inhibiting glycosphingolipid synthesis, but not catabolism, may be a therapeutic approach for treating lupus nephritis. Others have suggested this approach since the features of lupus nephritis are similar to other chronic kidney diseases characterized by altered glycosphingolipid metabolism (52, 104, 105).

Ozanimod (RPC1063), a modulator of S1P receptors 1 and 5, was evaluated in the lupus mouse model NZBWF1 as a potential treatment of lupus nephritis (106). The lupus-prone mice showed dose dependent improvement in lupus nephritis pathology when treated with ozanimod (0.3, 1.0, 3.0 mg/kg). There was a significant decrease in proteinuria (*p* < 0.01), mesangial expansion (*p* < 0.001), endocapillary proliferation (*p* < 0.0001), glomerular deposits (*p* < 0.0001), interstitial infiltrates (*p* < 0.0001), tubular atrophy (*p* < 0.01) and interstitial fibrosis (*p* < 0.01) in the 3.0 mg/kg ozanimod-treated mice when compared to the vehicle-treated control mice (106). Ozanimod significantly improved the kidney pathology related to lupus nephritis and chronic inflammation, demonstrating a need for further studies in human populations.

Recently, a group from Switzerland, who has been investigating the efficacy of cenerimod, an S1P receptor 1 modulator in Phase 2 development for treatment of SLE, in collaboration with Anaheim Clinical Trials (LLC, United States), examined the pharmacokinetics (PK), pharmacodynamics (PD), as well as safety and tolerability in Caucasian and Asian subjects to allow for more recruitment in future studies (107). The drug was found to be safe and well tolerated, and the team reported the absence of any relevant PK or PD differences in Caucasian and Asian patients, supporting the use of the same dose (single, oral dose of 4 mg) of cenerimod in upcoming late-phase studies.

In summary, evidence that supports the possible use of sphingolipids as a future screening tool has been presented in this review (summary of sphingolipids in human SLE studies is in Supplementary Table 1). As of yet sphingolipid tests lack the specificity and sensitivity to be used alone as a diagnostic tool for SLE. Nonetheless, clinical laboratories have started performing a diagnostic test that quantifies plasma levels of ceramides to identify patients at higher risk of developing major adverse cardiovascular events, which could be helpful for SLE patients at high risk of developing CVD. Several sphingolipid species were found to be important biomarkers in the prognosis, and organ-specific damage in SLE. Targeting sphingolipids and sphingolipid metabolism is a promising avenue to prevent progression of SLE and allow for better health outcomes in SLE patients. While several studies have been conducted on lupus-induced mice models, further studies are needed in the form of human trials.

## Author Contributions

Both authors made an extensive, precise, and intellectual contribution to the work. SH approved it for publication.

## Conflict of Interest

The authors declare that the research was conducted in the absence of any commercial or financial relationships that could be construed as a potential conflict of interest.

## Supplementary Material

Supplementary Table 1 Summary of plasma/serum and urine sphingolipids in systemic lupus erythematosus.

## Abbreviations

ANA: anti-nuclear antibody
APLA: antiphospholipid antibodies
Sm: anti-Smith
BILAG: British Isles Lupus Assessment Group Scale
CVD: cardiovascular disease
CNS: central nervous system
COPD: chronic obstructive pulmonary disease
CAD: coronary artery disease
dPE: diacyl phosphatidylethanolamine
dhS1P: dihydrosphingosine 1-phosphate
eNOS: endothelial nitric oxide synthase
pPEs: ethanolamine plasmalogens
Hex-Cer: hexosylceramide
iNOS: inducible nitric oxide synthase
IFN: interferon
Lact-Cer: lactosylceramide
PE: phosphatidylethanolamine
pDC: Plasmacytoid dendritic cell
SM: sphingomyelin
S1P: sphingosine 1-phosphate
SK: sphingosine kinase
SREBP: sterol regulatory element binding protein
SLAM: Systemic Lupus Activity Measurement
SLAM-R: Systemic Lupus Activity Measurement revised
SLE: systemic lupus erythematosus
SLEDAI: Systemic Lupus Erythematosus Disease Activity Index
SLEDAI-2K: Systemic Lupus Erythematosus Disease Activity Index revised in 2002
SLICC/ACR: Systemic Lupus International Collaborating Clinics Damage Index
TLR: toll-like receptor
IFN-1: type 1 Interferon.
