# Supplementary material for: Sphingolipids and Diagnosis, Prognosis, and Organ Damage in Systemic Lupus Erythematosus
Source: Front Immunol. 2020 Sep 25;11:586737. doi: 10.3389/fimmu.2020.586737 (PMC7546393; doi:10.3389/fimmu.2020.586737)
Supplement: Supplementary Table 1 — Summary of plasma/serum and urine sphingolipids in systemic lupus erythematosus. [file Table_1.pdf]

TABLE S1: Summary of plasma/serum and urine sphingolipids in Systemic Lupus Erythematosus

Sphingolipids	SLE *vs.* Ctl (plasma)	SLAM *vs.* Ctl (plasma)	SLEDAI *vs.* Ctl (plasma)	SLICC *vs.* Ctl (plasma)	SLAM ≥7 *vs.* SLAM <7 (plasma)	SLEDAI ≥6 *vs.* SLEDAI <6 (plasma)	Response to treatment (plasma)	MRL/lrp lupus mouse model Response to OP treatment (urine)	Lupus Nephritis *vs.* Ctl (serum)	Active renal disease *vs.* BILAG (plasma)	Lupus Nephritis *vs.* Ctl (urine)	Lupus Nephritis *vs.* Ctl (serum or plasma)	Lupus Nephritis *vs.* SLE without renal impairment (serum)	W SLE *vs.* W Ctl (plasma)	AA SLE *vs.* AA Ctl (plasma)	W & AA SLE *vs.* Ctl (plasma)	AA SLE Athero *vs.* W SLE Athero (plasma)
**Sphingomyelin**								↑ (103)									
C16:0							↓ (41)										
C24:1	↑ (41)																
C42:2	↑ (23)																
**Ceramide**								↑ (103)									
C16:0	↑ (23)<br>↑ (41)				↑ (41)	↑ (41)	↓ (41)					↑ (53)	↑ (53)		↑ (18)	↑ (18)	
C18:0	↑ (23)<br>↑ (41)						↓ (41)					↑ (53)	↑ (53)			↑ (18)	
C18:1														↑ (18)		↑ (18)	
C20:0	↑ (41)											↑ (53)	↑ (53)			↑ (18)	
C20:1														↑ (18)	↑ (18)	↑ (18)	
C22:0	↓ (17)						↓ (41)										
C23:0	↓ (17)																
C24:0	↑ (17)																
C24:1	↑ (41)	↑ (41)	↑ (41)	↑ (41)			↓ (41)					↑ (53)	↑ (53)				
C24:2	↑ (23)															↑ (18)	
C26:1															↑ (18)	↑ (18)	
**Lactosylceramide**								↑ (103)									
C16:0											↑ (51)						
C18:0																	↑ (18)
C20:0																	↑ (18)
**Hexosylceramides**								↑ (103)									
C16:0	↑ (41)	↑ (41)	↑ (41)	↑ (41)			↓ (41)			↑ (41)							
C18:0	↑ (41)						↓ (41)										
C18:1	↑ (41)																
C20:0															↑ (18)		
C20:1															↑ (18)		↑ (18)
C22:1															↑ (18)	↑ (18)	↑ (18)
C24:1	↑ (41)	↑ (41)	↑ (41)	↑ (41)			↓ (41)			↑ (41)					↑ (18)		
C26:0															↑ (18)		
C26:1															↑ (18)		
**Glucosylceramide**																	
C16:0							↓ (47)										
**Dihydroceramide**																	
C16:0	↑ (41)						↓ (47)<br>↓ (41)										

**Sphinganine (dihydrosphingosine)**												↑ (53)		↑ (18)		↑ (18)	↑ (18)
**sphingosine**	↓ (41)								↓ (50)			↑ (53)		↑ (18)	↑ (18)	↑ (18)	↑ (18)
**Sphinganine 1-phosphate**												↑ (53)		↑ (18)	↑ (18)	↑ (18)	
**Sphingosine 1-Phosphate (S1P)**	↓ (41)						↑ (41)					↑ (53)		↑ (18)		↑ (18)	
**Ceramide C16:0/S1P**		↑ (41)	↑ (41)	↑ (41)	↑ (41)	↑ (41)								↓ (18)		↓ (18)	
**Ceramide C24:1/S1P**		↑ (41)	↑ (41)	↑ (41)										↓ (18)		↓ (18)	

SLE: Systemic Lupus Erythematosus; SLAM: Systemic Lupus Activity Measurement; SLEDAI: Systemic Lupus Erythematosus Disease Activity Index; SLICC: Systemic Lupus International Collaborating Clinics Damage Index;
BILAG: British Isles Lupus Assessment Group Scale; W: White; AA: African American; Athero: Atherosclerosis; OP: Oseltamivir phosphate; Ctl: Control
Data presented are significant at a p value ≤0.05; empty cells denote statistically not significant or not measured
Numbers in brackets denote reference numbers
